# NiONP-Induced Oxidative Stress and Mitochondrial Impairment in an In Vitro Pulmonary Vascular Cell Model Mimicking Endothelial Dysfunction

**DOI:** 10.3390/antiox11050847

**Published:** 2022-04-26

**Authors:** Ophélie Germande, Thomas Ducret, Jean-Francois Quignard, Juliette Deweirdt, Véronique Freund-Michel, Marie-Hélène Errera, Guillaume Cardouat, Pierre Vacher, Bernard Muller, Patrick Berger, Christelle Guibert, Magalie Baudrimont, Isabelle Baudrimont

**Affiliations:** 1 Université de Bordeaux, 146, Rue Léo Saignat, F-33076 Bordeaux, France; ophelie.germande@u-bordeaux.fr (O.G.); thomas.ducret@u-bordeaux.fr (T.D.); jean-francois.quignard@u-bordeaux.fr (J.-F.Q.); veronique.michel@u-bordeaux.fr (V.F.-M.); guillaume.cardouat@u-bordeaux.fr (G.C.); bernard.muller@u-bordeaux.fr (B.M.); patrick.berger@u-bordeaux.fr (P.B.); 2 Inserm U 1045, Centre de Recherche Cardio-Thoracique, Avenue du Haut Lêveque, F-33604 Pessac, France; pierre.vacher@inserm.fr (P.V.); christelle.guibert@u-bordeaux.fr (C.G.); 3 Université de Bordeaux, CNRS, EPHE, UMR EPOC 5805, Place du Dr Peyneau, F-33120 Arcachon, France; 4 ANSES, Agence Nationale de Sécurité Sanitaire de L’alimentation de L’environnement et du Travail, Direction de L’Évaluation des Risques, Unité Évaluation des Substances Chimiques, F-94701 Maisons-Alfort, France; juliette.deweirdt@anses.fr; 5 Department of Ophtalmology, University of Pittsburgh School of Medecine, Pittsburgh, PA 15260, USA; errera.mhelene@gmail.com; 6 CHU de Bordeaux, Service d’Exploration Fonctionnelle Respiratoire, Service de Pédiatrie Médicale, F-33076 Bordeaux, France

**Keywords:** endothelial dysfunction, calcium, cyclic stretch, human pulmonary artery endothelial cells, nickel oxide nanoparticles, mitochondria alteration, reactive oxygen species

## Abstract

The development and use of nanomaterials, especially of nickel oxide nanoparticles (NiONPs), is expected to provide many benefits but also has raised concerns about the potential human health risks. Inhaled NPs are known to exert deleterious cardiovascular side effects, including pulmonary hypertension. Consequently, patients with pulmonary hypertension (PH) could be at increased risk for morbidity. The objective of this study was to compare the toxic effects of NiONPs on human pulmonary artery endothelial cells (HPAEC) under physiological and pathological conditions. The study was conducted with an in vitro model mimicking the endothelial dysfunction observed in PH. HPAEC were cultured under physiological (static and normoxic) or pathological (20% cycle stretch and hypoxia) conditions and exposed to NiONPs (0.5–5 μg/cm^2^) for 4 or 24 h. The following endpoints were studied: (i) ROS production using CM-H_2_DCF-DA and MitoSOX probes, (ii) nitrite production by the Griess reaction, (iii) IL-6 secretion by ELISA, (iv) calcium signaling with a Fluo-4 AM probe, and (v) mitochondrial dysfunction with TMRM and MitoTracker probes. Our results evidenced that under pathological conditions, ROS and nitrite production, IL-6 secretions, calcium signaling, and mitochondria alterations increased compared to physiological conditions. Human exposure to NiONPs may be associated with adverse effects in vulnerable populations with cardiovascular risks.

## 1. Introduction

Air quality is determined by the pollutants present in the atmosphere and in the air we breathe. In 2017, air pollution was implicated in up to 7 million premature deaths worldwide [1]. Additionally, human exposure to pollutants has risen as a result of an exponential increase in the use of nanoparticles (NPs) in industry, leading to a public health concern.

Many epidemiological studies have established correlations between the inhalation of ultrafine particle and higher incidences of human cardiovascular and pulmonary diseases [2,3,4]. Among the growing industries, nickel (Ni) production has increased in particular, due to the use of Ni in many areas of factory production, such as the production of catalysts, energy storage devices, ands lithium-ion batteries for electric cars [5]. New Caledonia is the world’s third largest Ni producer. Mineral resources are concentrated in ultramafic formations characterized by high concentrations of trace metals, including Ni, chromium (Cr), cobalt (Co), and manganese (Mn) [6,7]. Anthropic activities such as open-pit mining and the natural erosion of soils in New Caledonia has led to the atmospheric emission of Ni particles and, in particular, to the emission of nickel oxide nanoparticles (NiONPs) due to the hydrometallurgical process used to extract Ni [8,9]. Previous studies have revealed significant contamination levels in the New Caledonian population and have shown elevated levels of Ni in the lungs of nickel refinery workers [10,11]. Moreover, cardiovascular diseases were found to be more prevalent among those workers [12]. Only a few studies have assessed the risks for workers and the surrounding population, yet the risks of inhaled NiONPs to vulnerable populations suffering from chronic cardiovascular disease have not been documented. However, a study evaluated the effects of inhaled particulate matter of 2.5 µm in diameter (PM_2.5_) on a population suffering from chronic cardiovascular diseases [13]. NiONPs are potentially more harmful than PM_2.5_ because of their high surface reactivity, which leads to greater interactions with biological systems [14].

A previous study showed greater lung deposition with inhaled NPs than with larger-sized particles because inhaled NPs are able to accumulate in lung alveoli and in the lung parenchyma near the pulmonary arteries [15]. NPs can efficiently cross the pulmonary epithelial barrier to reach the pulmonary circulation, where they can be in direct contact with the endothelial cells (EC) that line the inner surface of the arteries [1,16]. Any alterations of EC can induce vascular diseases. Indeed, EC are involved in vascular tone regulation, coagulation control, and vascular barrier integrity [17]. Owing to their direct contact with the blood stream, EC are also very vulnerable to mechanical forces such as the shear stress and cyclic stretch (CS) resulting from frictional forces in the blood flow and intraluminal pressure [18,19]. In vivo studies in rodents have shown pulmonary inflammation and pulmonary fibrosis induced by NiONPs [20,21,22]. Both pulmonary inflammation and fibrosis are known to play a role in cardiovascular diseases [23]. Furthermore, we previously demonstrated, in an in vitro study of EC, that NiONPs trigger significant oxidative stress. This was shown through an overproduction of reactive oxygen species (ROS) associated with a pro-inflammatory response, mitochondrial dysfunction, and calcium signaling alterations [24], all critical events involved in the pathophysiology of cardiovascular diseases such as pulmonary hypertension (PH) [25,26]. Moreover, our findings suggest that NiONPs interact with mechanosensitive ion channel TRPV4 (transient receptor potential vanilloid 4), which is expressed in vascular cells and is also involved in the remodeling of pulmonary arteries, a major feature of PH [27,28]. We also showed that NiONPs induce an increase in vasoconstrictor mediator endothelin-1 (ET-1) production in EC, which is also involved in PH [29]. Consequently, NiONP accumulation and retention in pulmonary arteries could be a critical factor for the exacerbation of pre-existing cardiovascular diseases, such as PH, which is the main disease of the pulmonary circulation. This disease is characterized by an increase in pulmonary arterial resistance, a remodeling of pulmonary arteries, perivascular inflammation, pulmonary arterial hyper-reactivity with an elevation in pulmonary arterial pressure, right ventricular hypertrophy, and, finally, heart failure leading to premature death [30,31].

Moreover, all the critical events triggered by NiONPs, such as oxidative stress, inflammation, and calcium signaling alterations, are key features of PH pathophysiology. Thus, one can assume that the workers and the population exposed to NiONPs in New Caledonia may be at risk of exacerbating preexisting cardiovascular diseases such as PH. However, the underlying cellular and molecular mechanisms by which NiONPs alter both calcium signaling and mitochondrial function in human pulmonary artery endothelial cells (HPAEC) under pathological conditions have not been reported. In this respect, the objectives of the proposed in vitro study are to compare these NiONP-dependent mechanisms in HPAEC cultured under physiological and pathological conditions. These conditions mimic the vascular dynamics and the environment observed in PH [32].

Different endpoints were investigated in both conditions: (i) ROS production was investigated using CM-H_2_DCF-DA and MitoSOX probes, (ii) nitrite production by the Griess reaction, (iii) IL-6 secretion by ELISA, (iv) calcium signaling by Fluo-4 AM probe, and (v) mitochondrial dysfunction with TMRM and MitoTracker probes.

## 2. Materials and Methods

### 2.1. Human Pulmonary Arterial Endothelial Cell Culture

Human pulmonary arterial endothelial cells (HPAEC) obtained from the main branch of the pulmonary artery of a 68-year-old Caucasian female donor (N°451Z031.14) were provided by PromoCell^®^ (Heidelberg, Germany). HPAEC were cultured in Endothelial Cell Growth Medium (ECGM) supplemented with Supplement Mix, as recommended by the manufacturer (PromoCell^®^). Cells were seeded at 20,000 cells/cm^2^ in 25 cm^2^ culture flasks and were cultured at 37 °C, in 95% humidity and 5% CO_2_. Cell passages were conducted when cells were at about 80% confluence. All experiments were conducted on HPAEC from passages 2 to 8.

### 2.2. Reagents and Chemicals

HPAEC were cultured in ECGM from PromoCell^®^. The standard physiological salt solution (PSS) was composed of 130 mM NaCl, 5.6 mM KCl, 8 mM HEPES, 11 mM Glucose, 1 mM Mg^2+^, and 2 mM Ca^2+^ adjusted to pH = 7.4. Fluo-4-AM green dye (1 µM), MitoSOX^TM^ red dye (5 µM), and Hoechst 33342 blue dye (2 µM) were obtained from Thermo Fischer Scientific, Invitrogen™ (Paris, France).

### 2.3. NiO Nanoparticles

Nickel (II) oxide nanoparticles (NiONPs) (Cat. No.637130) were obtained from Sigma-Aldrich (St. Louis, MO, USA). NiONPs partially characterized by the manufacturer have the following physical and chemical characteristics: mean aerodynamic diameter of 50 nm (TEM); density, 6.67 g/mL; purity, 99.8% (trace metal basis); refractive index, 1.331; and viscosity, 0.888 cP. NiONPs were suspended in ECGM at a final concentration of 2 mg/mL and stored at 4 °C until use. Prior to use, stock suspensions were vortexed, sonicated at 3 × 30 s (Vibracell 75186, 130 W, 56–60 Hz), and diluted in ECGM extemporaneously at appropriate concentrations (0.5–5 µg/cm^2^). The NiONPs were previously characterized by Germande et al., 2022 [24]. The hydrodynamic size distribution of NiONPs suspended in ECGM was determined at a concentration of 5 µg/cm^2^ by dynamic light scattering (DLS) using a VASCO particle-size analyzer from Cordouan Technologies^®^ (Pessac, France). The Zeta potential values of NiONPs were assessed by laser Doppler electrophoresis and a Wallis zeta-potential analyzer from Cordouan Technologies^®^.

### 2.4. Acellular ROS Production

Acellular ROS production was assessed using the H_2_DCF-DA probe (Fisher Scientific^®^), as recommended by the manufacturer and using a method adapted from previous studies [33]. The H_2_DCF-DA probe was resuspended with a mix of absolute ethanol (final concentration 0.33 mM) and a 10 mM NaOH solution to cleave the diacetate group. After a 30 min activation at room temperature in the dark, the reaction was stopped with 10× PBS. The activated H_2_DCF probe was then incorporated (final concentration 16.5 μM) with NiONPs (0–50 µg/mL). Finally, the plate was incubated for 60 min at 37 °C and 5% CO_2_. Fluorescence intensity was measured by spectrofluorimetry at 485/520 nm (excitation/emission) by using the FLUOstar Omega 2.10 plate reader, and the analyses were performed with MARS Data Analysis Software 2.30 R3 (BMG Labtech^®^, Ortenberg, Germany).

### 2.5. Cyclic Stretch

After 48 h of culture in flexible silicon chambers, cells were placed under either static and normoxic conditions (21% O_2_, 37 °C, and 5% CO_2_) or under 20% cyclic stretch (CS) (1 Hz frequency) and hypoxic conditions (1% O_2_, 37 °C, and 5% CO_2_) for 20 h using a STREX^®^ system ST-140-10 (B-bridge international, Santa Clara, CA, USA). After 20 h, cells and supernatants were collected.

### 2.6. Nanoparticle Exposure

After 20 h of culture in both physiological (static cells in normoxia) and pathological (20% CS in hypoxia) conditions, cells were treated with NiONPs (0.5–5 µg/cm^2^) for 4 h or 24 h. These different durations of HPAEC exposure to NiONPs were chosen according to various kinetic studies which showed a peak in ERO production after 4 h of exposure of HPAEC to NiONPs and peaks in IL-6 and nitrite secretion after 24 h of exposure of cells to NPs (results not shown). Supernatants or cells were then collected to perform the experiments. The NiONPsworking concentrations for all experiments (for which the rate of mortality was under 30%) were all determined previously with the WST-1 assay test [24].

### 2.7. Oxidative Stress

Cells were cultured at 20,000 cells/cm^2^ and maintained for 48 h in both physiological and pathological conditions. Cells were then exposed to NiONPs (0.5–5 µg/cm^2^) for 4 h.

#### 2.7.1. Global Reactive Oxygen Species Production

Intracellular ROS production was performed using the CM-H_2_DCF-DA probe (Fisher Scientific^®^), as recommended by the manufacturer and using a method adapted from previous studies [33]. Cells were rinsed with ECGM without serum and were pre-incubated for 20 min with CM-H_2_DCF-DA probe (final concentration 20 µM) before a 4 h exposure to NiONPs. Fluorescence intensity was measured by spectrofluorimetry at 485/520 nm (excitation/emission) by using the FLUOstar Omega 2.10 plate reader (BMG Labtech^®^, Champigny s/Marne, France), and the analyses were performed with MARS Data Analysis Software 2.30 R3 (BMG Labtech^®^).

#### 2.7.2. Mitochondrial O_2_^−^ production

Mitochondrial superoxide anion (O_2_^−^) formation was performed using MitoSox^TM^ red dye (ThermoFischer^®^), as recommended by the manufacturer by confocal microscopy (TE 2000, Nikon). After a 4 h exposure to NiONPs, cells were incubated for 30 min (37 °C, in the dark) with MitoSox probe in PSS. The MitoSox probe was co-incubated with a nucleus probe (Hoechst 33342). Fluorescence intensity was measured for MitoSox at 543/605 nm and Hoechst 33342 at 408/450 nm (excitation/emission). The silicon chambers were observed at 40× magnification with an oil immersion objective on a laser scanning confocal microscope (TE 2000, Nikon, Tokyo, Japan). For each experiment, 20–30 cells were analyzed per well. MitoSox fluorescence intensity was proportional to the rate of probe oxidation. Analyses were performed using NIS-Elements AR software 3.0 and Microsoft Office Excel 2016 (Redmond, WA, USA).

### 2.8. Nitric Oxide Metabolite Production: Nitrites

Cells were cultured at 20,000 cells/cm^2^ and maintained for 48 h in both physiological and pathological conditions. Cells were then exposed to NiONPs (0.5–5 µg/cm^2^) for 24 h. The presence of nitrite products was determined in HPAEC supernatants with Griess reagent (Sigma-Aldrich^®^), as recommended by the manufacturer. After a 24 h exposure to NiONPs, the supernatants were removed, collected, and centrifuged at 10,000× *g* for 10 min at 4 °C. Then, the supernatants were analyzed as recommended by the manufacturer’s guidelines and the absorbance was measured at 540 nm by spectrophotometry using a microplate spectrophotometer reader (SPECTROstarNano2.10, BMG Labtech^®^).

### 2.9. Pro-Inflammation Effect: Cytokine IL-6 Production

The level of pro-inflammatory interleukin-6 (IL-6) in supernatants was assessed and analyzed using an ELISA kit (Human IL-6 DuoSet^®^ ELISA R&D Systems, Minneapolis, MA, USA). Cells were cultured at 20,000 cells/cm² and maintained for 48 h in both physiological and pathological conditions. Cells were washed with the culture medium and exposed to NiONPs (0.5–5 µg/cm²) for 24 h. Supernatants were then removed, centrifuged at 10,000× *g* for 10 min at 4 °C, and analyzed as recommended by the manufacturer. The absorbance was determined at 450 nm, corrected at 570 nm by spectrophotometry using a microplate spectrophotometer reader (SPECTROstarNano 2.10, BMG Labtech^®^).

### 2.10. Cytoplasmic Calcium Measurement

Variations in intracellular calcium [Ca^2+^]_c_ were detected using the Fluo-4 AM green dye, as recommended by the manufacturer’s guidelines. Cells were cultured at 20,000 cells/cm^2^ and maintained for 48 h in both physiological and pathological conditions. After a 4 h exposure to NiONPs (0.5–5 µg/cm^2^), cells were incubated for 30 min (37 °C, in the dark) with the Fluo-4 AM probe in PSS. Fluo-4-AM probe was co-incubated with a nucleus probe (Hoechst 33342). The silicon chambers were observed at 40× magnification with an oil immersion objective on a laser scanning confocal microscope (TE 2000, Nikon). Fluorescence intensity was determined for Fluo-4 AM at 488/515 nm and for Hoechst 33342 at 408/450 nm (excitation/emission). For each experiment, 20–30 cells were analyzed per well. The analyses were performed using NIS-Elements AR software 3.0 and Microsoft Office Excel.

### 2.11. Mitochondrial Activity

#### 2.11.1. Mitochondrial Membrane Potential Measurement

The mitochondrial membrane potential (ΨMP) was measured by confocal microscopy, using TMRM red dye (Tetramethylrhodamine, Methyl Ester, Perchlorate, ThermoFischer) as recommended by the manufacturer’s guidelines. Cells were cultured at 20,000 cells/cm^2^ and maintained for 48 h in both physiological and pathological conditions. After a 4 h exposure to NiONPs (0.5–5 µg/cm^2^), cells were incubated for 20 min (37 °C, in the dark) with a TMRM fluorescent probe in PSS. The TMRM probe was co-incubated with a nucleus probe (Hoechst 33342). The silicon chambers were observed at 40× magnification with an oil immersion objective on a laser scanning confocal microscope (TE 2000, Nikon). The Fluorescence intensity was then determined at 543/605 nm (excitation/emission). For each experiment, 20–30 cells were analyzed per well. The analyses were performed using the NIS-Elements AR software 3.0 and Microsoft Office Excel.

#### 2.11.2. Mitochondrial Mass

The mitochondrial mass was measured by confocal microscopy using the MitoTracker green dye (ThermoFischer) as recommended by the manufacturer’s guidelines. Cells were cultured at 20,000 cells/cm^2^ and maintained for 48 h in both physiological and pathological conditions. After a 4 h exposure to NiONPs (0.5–5 µg/cm^2^), cells were incubated for 30 min (37 °C, in the dark) with the MitoTracker probe in PSS. The MitoTracker probe was co-incubated with a nucleus probe (Hoechst 33342). The silicon chambers were observed at 40× magnification with an oil immersion objective on a laser scanning confocal microscope (TE 2000, Nikon). The Fluorescence intensity was determined at 408/450 nm (excitation/emission). For each experiment, 20–30 cells were analyzed per well. The analyses were performed using the NIS-Elements AR software 3.0 and Microsoft Office Excel.

### 2.12. Statistical Analysis

Every experimental condition was independently repeated several times (*n* indicates the number of experiments) and for all independent experiments, 3–8 wells per condition were conducted. Data are expressed as mean ± standard error of the mean (SEM) for *n* independent experiments. Statistical tests were assessed using analysis of variance: one-way ANOVA followed by Tukey’s post hoc test for multiple comparisons (*n* > 30) and by Kruskal–Wallis test followed by Dunn’s post hoc test (*n* < 30). (* *p* < 0.05, ** *p* < 0.01 and *** *p* < 0.001 vs. untreated cells in 0% CS in normoxic conditions. # *p* < 0.05, ## *p* < 0.01 and ### *p* < 0.001 vs. untreated cells in pathological conditions. $ *p* < 0.05, $$ *p* < 0.01 and $$$ *p* < 0.001 between both conditions). All data were analyzed with GraphPad PRISM software. *p* values < 0.05 were considered statistically significant.

## 3. Results

### 3.1. Acellular ROS Production and Morphological Study of HPAEC in Physiological and Pathological Conditions

#### 3.1.1. Acellular ROS Production in Cell Medium

**Figure 1:**
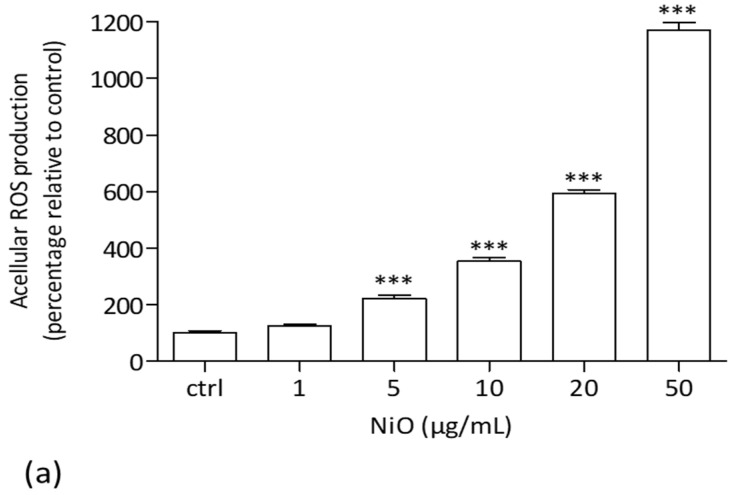

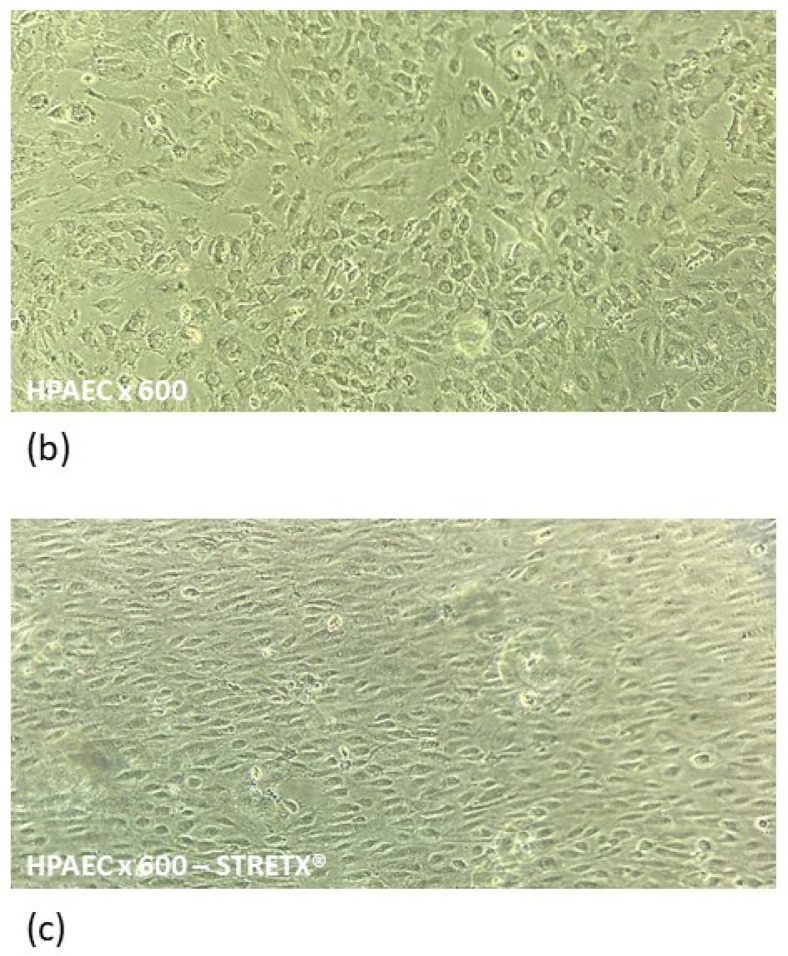
(**a**) Acellular NiONP ROS production measured by spectrofluorimetry with the H2DCF-DA probe. The results were expressed as the H2DCF probe fluorescence intensity relative to controls (medium without NiONPs). *** *p* < 0.001 vs. control according to Kruskal–Wallis test followed by a Dunn’s multiple comparison test. (**b**,**c**) HPAEC after 20 h of culture, either under physiological or pathological conditions, observed by phase contrast microscopy (PCM) at 600× magnification. (**b**) Untreated cells under static conditions in normoxia. (**c**) Untreated cells under 20% CS in hypoxia.

In acellular conditions, NiONPs were able to induce ROS generation in the culture medium. We observed from 5 µg/mL a significant concentration-dependent increase in global ROS production as compared to controls, i.e., medium without NiONPs (122.85%, *** *p* < 0.001) (Figure 1a).

#### 3.1.2. Morphological Study of HPAEC

HPAEC were first observed by phase contrast microscopy (PCM). Microscopic images were obtained after 20 h under physiological and pathological conditions. The representative images shown in Figure 1b,c demonstrate that the morphology of cells cultured under 20% CS was more elongated as compared to static cultured cells.

### 3.2. NiONP-Induced Oxidative Stress under Physiological and Pathological Conditions

To investigate how CS and hypoxia modulate ROS production, measurements of global ROS and mitochondrial O_2_^−^ production were performed.

#### 3.2.1. Cytoplasmic ROS Production

**Figure 2:**
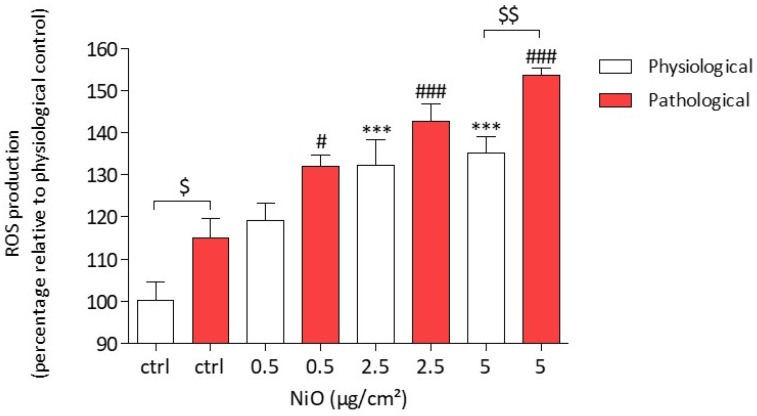
ROS production in HPAEC after a 4 h exposure to NiONPs (0.5–5 μg/cm^2^) under physiological and pathological conditions, as measured by spectrofluorimetry with the CMH_2_DCF-DA probe. The results were expressed as the percentage of CMH_2_DCF probe fluorescence intensity relative to physiological controls. Data were mean ± SEM of three independent experiments (*n* = 3) performed in quadruplicate. *** *p* < 0.001 vs. untreated cells in 0% CS in normoxic conditions. # *p* < 0.05 and ### *p* < 0.001 vs. untreated cells in pathological conditions. $ *p* < 0.05 and $$ *p* < 0.01 between both conditions. According to Kruskal–Wallis test followed by a Dunn’s multiple comparison test.

After a 4 h exposure of HPAEC to NiONPs (0.5–5 µg/cm^2^) under physiological conditions, we observed from 2.5 µg/cm^2^ a significant concentration-dependent increase in global ROS production as compared to untreated cells (132.29 %, # *p* < 0.05). Similarly, under pathological conditions, global ROS production was also significantly higher in a concentration-dependent pattern, but from 0.5 µg/cm^2^, as compared to untreated cells (132.08 %, *** *p* < 0.001). However, under pathological conditions, for the highest NiONP concentration, global ROS production was significantly increased as compared to treated cells under physiological conditions (135.21 % vs. 153.57 %, $$ *p* < 0.01), demonstrating that ROS production induced by NiONP exposure was higher under pathological conditions than under physiological ones (Figure 2).

#### 3.2.2. Mitochondrial O_2_^−^ Production

**Figure 3:**
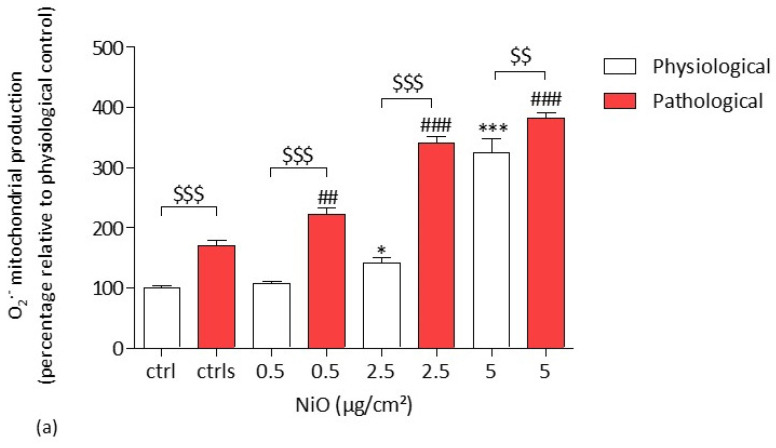

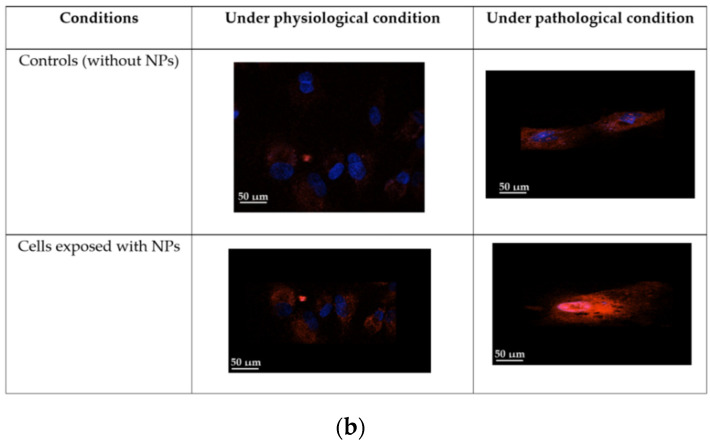
Mitochondrial O_2_**^−^** production in HPAEC after a 4 h exposure to NiONPs (0.5–5 μg/cm^2^) under physiological and pathological conditions, measured by confocal microscopy. (**a**) The values were normalized to the untreated physiological cells and results were expressed as the percentage of the MitoSox probe fluorescence intensity relative to the control cells. (**b**) HPAEC observed by confocal microscopy at focus 60×, under physiological and pathological conditions, without or with NiONPs (2.5 µg/cm²), with mitochondria marked in red, with MitoSox probe and nucleus in blue, with Hoechst probe. Data were mean ± SEM of three independent experiments (*n* = 3) performed in quadruplicate. * *p* < 0.05 and *** *p* < 0.001 vs. untreated cells in 0% CS in normoxic conditions. ## *p* < 0.01 and ### *p* < 0.001 vs. untreated cells in pathological conditions. $$ *p* < 0.01 and $$$ *p* < 0.001 between both conditions. According to one-way ANOVA followed by Tukey’s post hoc test for multiple comparisons.

After a 4 h exposure of HPAEC to NiONPs (0.5–5 µg/cm^2^), we observed a significant concentration-dependent increase in mitochondrial O_2_^−^ production at the two highest concentrations under physiological conditions (142.45% * *p* < 0.05 and 324.71 % *** *p* < 0.001) and from 0.5 µg/cm^2^ (222.35% ## *p* < 0.01) under pathological conditions as compared to untreated physiological cells, respectively (Figure 3a,b). Moreover, under pathological conditions, mitochondrial O_2_^−^ production was significantly increased as compared to untreated physiological cells (101% vs. 170.09% $$$ *p* < 0.001). Furthermore, under pathological conditions, for all the concentrations, mitochondrial O_2_^−^ production was significantly increased in response to NiONPs as compared to treated cells in physiological conditions ($$ *p* < 0.01 and $$$ *p* < 0.001). Figure 3b shows HPAEC under physiological or pathological conditions after NiONP exposure or not.

These results thus provide evidence that mitochondrial O_2_^−^ production was higher under pathological conditions after NiONP exposure as compared to physiological ones.

### 3.3. NiONP-Induced Nitrites Production under Physiological and Pathological Conditions

We have previously shown that after a 4 h exposure, NiONPs are able to increase intracellular O_2_^−^ production in HPAEC. Since NO can be oxidized by O_2_^−^ to form NO metabolites such as peroxynitrites (ONOO^−^), the production of nitrites was investigated as a marker of NO metabolites. The production of nitrites was measured using the Griess reaction. After a 24 h exposure of HPAEC to NiONPs (0.5–5 µg/cm^2^) under physiological conditions, a significant increase in nitrite production was observed at the highest concentration (0.18 ng/µL, * *p* < 0.05) as compared to untreated cells (0.09 ng/µL). Similarly, under pathological conditions, NiONPs induced a significant increase in nitrite production at 5 µg/cm^2^, as compared to untreated cells (0.32 ng/µL, # *p* < 0.05 and 0.20 ng/µL, respectively).

**Figure 4:**
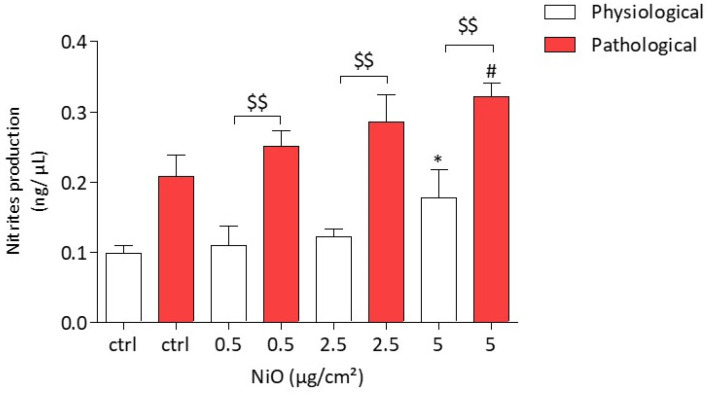
Nitrite production in HPAEC after a 24 h exposure to NiONPs (0.5–5 μg/cm^2^) under physiological and pathological conditions, measured by Griess reaction. The results were expressed as ng/µL in the supernatant. Data are mean ± SEM of five independents experiments (*n* = 5) performed in quadruplicate. * *p* < 0.05 vs. untreated cells in 0% CS in normoxic conditions. # *p* < 0.05 vs. untreated cells in pathological conditions. $$ *p* < 0.01 between both conditions. According to Kruskal–Wallis test followed by a Dunn’s multiple comparison test.

Interestingly, at the three NiONP concentrations, the production of nitrites under pathological conditions was significantly higher than for cells under physiological conditions ($$ *p* < 0.01) (Figure 4).

### 3.4. NiONP-Induced IL-6 Secretion under Physiological and Pathological Conditions

**Figure 5:**
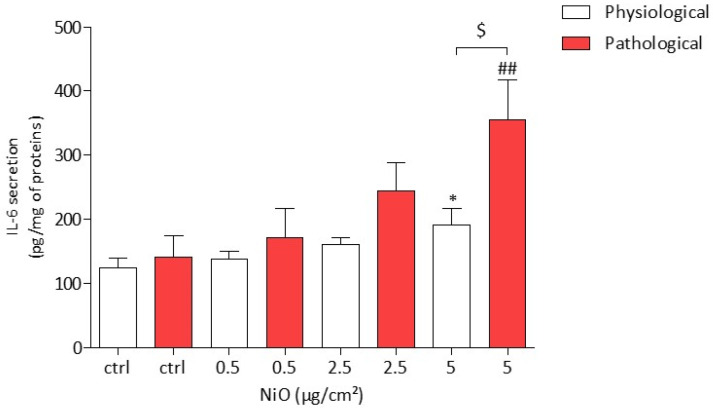
IL-6 production in HPAEC after a 24 h exposure to NiONPs (0.5–5 μg/cm^2^) under physiological and pathological conditions measured by ELISA. The results were expressed as pg/mg of protein. Data are mean ± SEM of five independents experiments (*n* = 5) performed in quadruplicate. * *p* < 0.05 vs. untreated cells in 0% CS in normoxic conditions. ## *p* < 0.01 vs. untreated cells in pathological conditions. $ *p* < 0.05 between both conditions. According to Kruskal–Wallis test followed by a Dunn’s multiple comparison test.

The IL-6 secretion was measured in HPAEC by ELISA assay. After a 24 h exposure of HPAEC to NiONPs (0.5–5 µg/cm^2^), the results showed, at 5 µg/cm^2^, a significant increase in IL-6 production under both physiological and pathological conditions (191.74 pg/mg of proteins * *p* < 0.05 and 355.14 pg/mg of proteins ## *p* < 0.01, respectively) as compared to untreated cells (124.17 pg/mg of proteins and 141.96 pg/mg, respectively) (Figure 5). Interestingly, at the highest concentration, 5 µg/cm², the secretion of IL-6 under pathological conditions was larger than that in physiological conditions ($ *p* < 0.05).

### 3.5. NiONP-Induced Cytosolic Calcium Level Alteration under Physiological and Pathological Conditions

**Figure 6:**
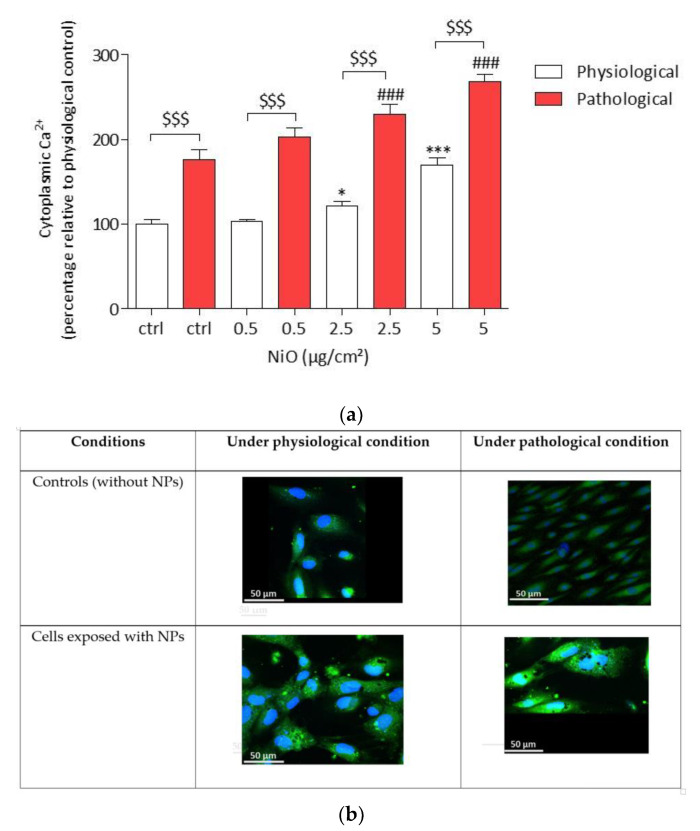
Resting [Ca^2+^]_c_ in HPAEC cells after a 4 h exposure to NiONPs (0.5–5 μg/cm^2^) in physiological and pathological conditions, measured with Fluo-4 probe (1 µM) by confocal microscopy. (**a**) The values were normalized to the untreated physiological cells and results were expressed as the percentage of the Fluo-4-AM probe fluorescence intensity relative to the physiological controls. (**b**) HPAEC observed by confocal microscopy at focus ×60, under physiological and pathological conditions, without or with NiONPs (5 µg/cm^2^), with cytoplasmic calcium marked in green with Fluo-4-AM probe and nucleus in blue with Hoechst probe. Data were mean ± SEM of three independents experiments (*n* = 3) performed in quadriplicate. * *p* < 0.05 and *** *p* < 0.001 vs. untreated cells in 0% CS in normoxic conditions. ### *p* < 0.001 vs. untreated cells in pathological conditions. $$$ *p* < 0.001 between both conditions. According to one-way ANOVA followed by Tukey’s post-test for multiple comparisons.

Variations in cytoplasmic calcium [Ca^2+^]_c_ were measured in HPAEC. After a 4 h exposure to NiONPs (0.5–5 µg/cm^2^), calcium imaging was assessed with Fluo-4 AM probe. The results showed a significant increase in cytoplasmic calcium levels from 2.5 µg/cm^2^ (121.53% * *p* < 0.05 and 230.06% ### *p* < 0.001) for both physiological and pathological conditions as compared to untreated cells (102.01% and 175.99%, respectively) (Figure 6a,b). Moreover, under pathological conditions, NiONPs-induced increase in basal [Ca^2+^]_c_ was higher than in physiological conditions whatever the concentration (0.5–5 µg/cm²) ($$$ *p* < 0.001). Figure 6b shows HPAEC under physiological or pathological conditions after NiONPs exposition or not.

### 3.6. NiONPs-Induced Mitochondria Alterations under Physiological and Pathological Conditions

#### 3.6.1. The Mitochondrial Membrane Potential

**Figure 7:**
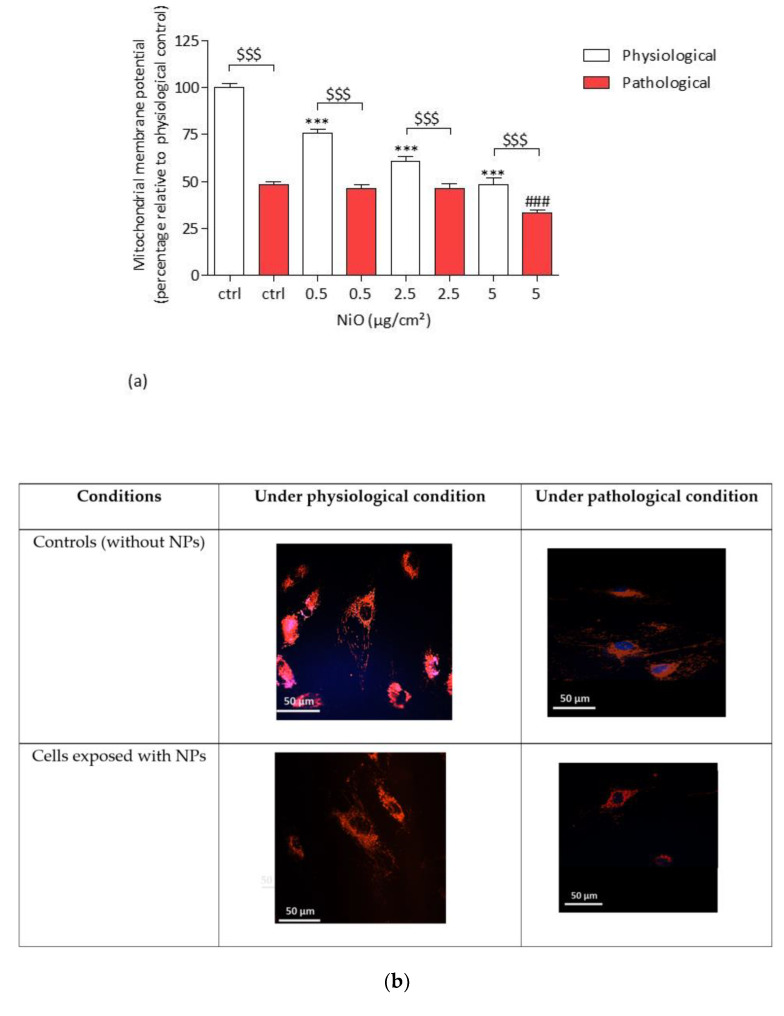

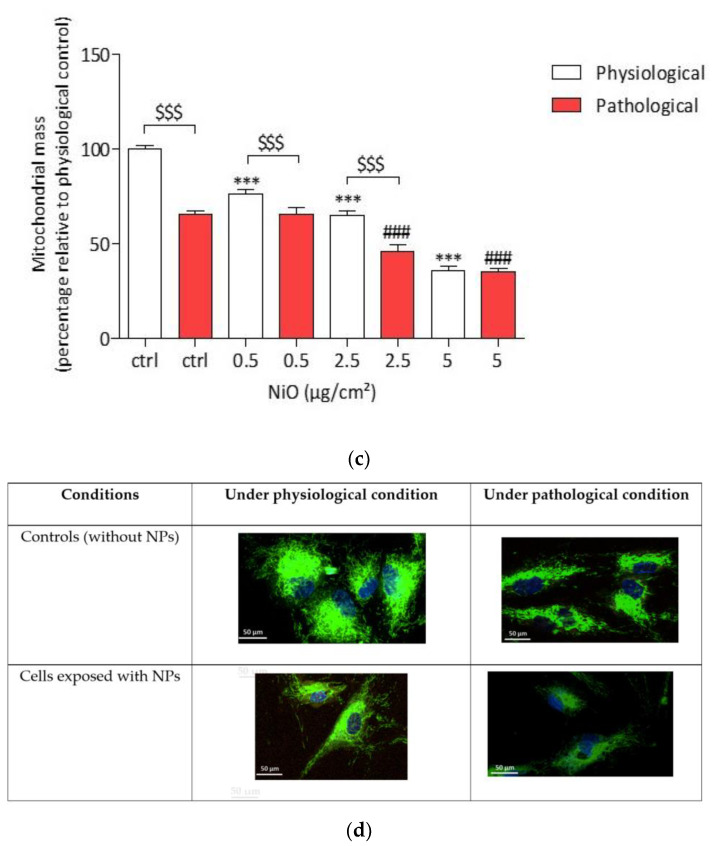
(**a**) ΨMP in HPAEC after a 4 h exposure to NiONPs (0.5–5 μg/cm^2^) in physiological and pathological conditions was measured with TMRM probe (100 nM) by confocal microscopy. (**b**) HPAEC observed by confocal microscopy at focus 60×, under physiological and pathological conditions, without or with NiONPs (5 µg/cm^2^), with ΨMP marked in red with TMRM probe and nucleus in blue with Hoechst probe. (**c**) Mitochondrial mass in HPAEC after a 4 h exposure to NiONPs (0.5–5 μg/cm^2^) in physiological and pathological conditions was measured with MitoTracker probe (1 µM) by confocal microscopy. The values were normalized to the untreated physiological cells and results were expressed as the fold change of the probes fluorescence intensity relative to the control cells. (**d**) HPAEC observed by confocal microscopy at focus 60×, under physiological and pathological conditions, without or with NiONPs (5 µg/cm^2^), with mitochondrial mass marked in green with MitoTracker probe and nucleus in blue with Hoechst probe. Data were mean ± SEM of three independents experiments (*n* = 3) performed in quadriplicate. *** *p* < 0.001 vs. untreated cells in 0% CS in normoxic conditions. ### *p* < 0.001 vs. untreated cells in pathological conditions. $$$ *p* < 0.001 between both conditions. According to one-way ANOVA followed by Tukey’s post hoc test for multiple comparisons.

After a 4 h exposure of HPAEC to NiONPs (0.5–5 µg/cm^2^), we observed a significant concentration-dependent decreased in the TMRM probe fluorescence resulting in a loss of mitochondrial membrane potential (ΨMP) in both physiological and pathological conditions as compared to untreated cells (Figure 7a,b). Under pathological conditions the loss of ΨMP was significantly observed only at 5 µg/cm^2^ (33.43% ## *p* < 0.01) and from the lowest concentrations in physiological conditions (75.57% *** *p* < 0.001 at 0.5 µg/cm^2^) as compared to untreated cells (99.99% under physiological conditions and 48.16% in pathological condition). Furthermore, under pathological conditions, a loss of ΨMP was significantly observed as compared to cells under physiological conditions ($$$ *p* < 0.001) for both treated and untreated cells. Figure 7b shows HPAEC under physiological or pathological conditions after NiONP exposure or not.

#### 3.6.2. The Mitochondrial Mass

After a 4 h exposure of HPAEC to NiONPs (0.5–5 µg/cm^2^), we observed a significant concentration-dependent decrease of mitochondrial mass in both physiological and pathological conditions as compared to untreated cells (Figure 7c,d), from 0.5 µg/cm^2^ (76.20% *** *p* < 0.001) in physiological conditions and from 2.5 µg/cm^2^ (46.10% ## *p* < 0.01) in pathological conditions. Moreover, under pathological conditions mitochondrial mass significantly decreased as compared to cells under physiological conditions in untreated cells (65.30% vs. 100.01%, respectively) and at the two lowest NiONP concentrations ($$$ *p* < 0.001). Figure 7d shows HPAEC under physiological or pathological conditions after NiONP exposure or not.

## 4. Discussion

Nickel production in New Caledonia, resulting from mining activities, releases NiONPs into the atmosphere, which could affect the health of workers and surrounding populations, especially those affected by a pre-existing cardiovascular disease. Indeed, it is well known that NPs can induce adverse effects in vascular reactivity, blood pressure, and endothelial function, which in turn leads to endothelial dysfunction and cardiovascular diseases [34,35]. Patients suffering from vascular disease such as PH could be at risk because pulmonary circulation, and especially ECs, are possible primary targets of inhaled NPs [36]. EC are subjected to hemodynamic forces, including shear stress and CS, which are increased in pulmonary arteries affected by PH [37]. Moreover, previous studies have shown that ECs are exposed to hypoxia, leading to endothelial dysfunction in hypoxemic PH [38]. Our study focused on oxidative stress, nitrite production, inflammation, calcium homeostasis, and mitochondrial function because many studies have shown that these factors are involved in the development and the progression of cardiovascular diseases such as PH [39] and altered in pulmonary cells exposed to NPs [40,41,42].

In this context, we aimed to evaluate whether NiONP exposure could aggravate vascular diseases such as PH. In an in vitro study, we compared the toxic effects of NiONPs n pulmonary vascular cells under physiological and pathological conditions. These conditions mimic the vascular dynamics and environment observed in PH, i.e., hypoxia and 20% CS. In a previous study, we showed that this experimental in vitro model closely reproduces in vivo PH events such as endothelial failure [32].

First, we showed that, in acellular conditions, ROS were generated in the culture medium by NiONPs, mainly due to their high surface reactivity. These results are in line with the literature [43]. The cytotoxicity of metal oxide NPs could therefore be due, in part, to the overproduction of ROS, and to their high surface reactivity [44,45]. Moreover, transition metals such as Ni are known to produce ROS such as O_2_^−^ via the Fenton reaction [46].

Dysregulation of the redox system disrupts the cellular signaling pathways involved in the development and progression of pulmonary vascular diseases. In addition, as EC are subjected to mechanical forces, they can react to these forces by changing their redox state, leading to pathophysiological consequences. Thus, any factor, such as NP exposure, that disrupts the redox system can therefore contribute to the aggravation or development of vascular diseases [47]. Oxidative stress is therefore a major event implicated in the pathophysiology of PH and in the vascular effects induced by NPs [48]. In the present study, we first demonstrated that a higher ROS production was observed in HPAEC under pathological conditions as compared to cells under physiological conditions. These results are in line with previous studies showing that hypoxia induces ROS production and endothelial dysfunction [30,31,32]. In addition, we showed that NiONP exposure significantly increased global ROS and mitochondrial O_2_^−^ production as compared to control cells. These results are in line with previously published data regarding NiONP exposure [24]. However, in cells exposed to NiONPs under pathological conditions, the increase in ROS and mitochondrial O_2_^−^ production was greater than in HPAEC under physiological conditions. These results suggest that the combination of both pathological conditions and NiONP exposure can result in higher oxidative stress, and can therefore lead to more severe endothelium damage.

It is well known that once ROS are produced, the unstable O_2_^−^, can react with the nitrous oxide (NO), an important vasculoprotective factor, to form reactive metabolites such as peroxynitrites and nitrites, toxic molecules implicated in endothelial dysfunction [49]. In a previous study, we showed that NiONPs induced a decrease in NO production in HPAEC [24] that could be explained by this rapid kinetic reaction between NO and O_2_^−^ leading to alterations of in vascular reactivity, one of the critical events observed in cardiovascular diseases, including PH [50,51]. Consequently, the production of nitrites, which reflects the peroxynitrites production, was analyzed. In the present study, NiONPs significantly increased, at the highest concentration, nitrite production in both physiological and pathological conditions. Moreover, in cells exposed to NiONPs under pathological conditions, the increase in nitrite production was greater than in HPAEC under physiological conditions, confirming the amplification of the toxic effects induced by NiONPs. These results are in line with previous studies showing a decrease in NO bioavailability associated with nitrite production under pathological conditions [52].

In view of the link between NP-induced oxidative stress and inflammation and the involvement of inflammation in the pathophysiology of PH [53], we also evaluated the effect of NiONPs on IL-6 secretion in physiological and pathological conditions. Indeed, it has been demonstrated both in vivo and in vitro that many metal oxide NPs induce ROS production, which could in turn activate inflammatory pathways via the secretion of pro-inflammatory cytokines, such as IL-6, [54]. IL-6 is a cytokine known to be involved in PH [55]. Our results showed that in NiONP–treated cells, IL-6 secretion is significantly higher when cells are cultured with 20% CS in hypoxia as compared to cells under physiological conditions. The increase in IL-6 secretion may reflect the pro-inflammatory response that can be found in PH [56].

Given the importance of calcium signaling in endothelial dysfunction, we also evaluated the effect of NiONP exposure on calcium signaling in physiological and pathological conditions. In a previous study, we demonstrated that NiONP-induced calcium homeostasis alterations may originate from oxidative stress [24]. Our findings suggested that calcium signaling alterations were partly due to both interaction and inactivation of TRPV4 calcium channels by NiONPs, which are known to have a major role in PH [57]. In the present study, it was observed that NiONPs increased basal variation [Ca^2+^]_c_ in HPAEC under both conditions. The present results are in line with those found in our previous study under physiological conditions [24]. In pathological conditions, it was observed that NiONPs induced basal cytoplasmic calcium release to a greater extent under pathological conditions as compared to physiological conditions, confirming a potential exposure risk for these NiONPs for populations that suffer from endothelial dysfunction.

All these results are in agreement with previous data showing that smaller and non-metallic, black carbon NPs induced endothelial dysfunction in HPAEC cultured on the same pathological model. This was characterized by an overproduction of ROS and nitrites, a secretion of pro-inflammatory IL-6 cytokine, and an alteration in calcium homeostasis [32].

In a previous study, we showed in HPAEC that NiONPs were rapidly internalized in the cytoplasm into vesicles to form aggregates which were detected very close to mitochondria, suggesting that they may be able to interfere with mitochondrial functions [24]. Moreover, in the current study, we demonstrated that NiONP exposure induced an overproduction of mitochondrial O_2_^−^. Li et al. [58] also showed that exposure to metal NPs (silver NPs) can alter mitochondria in relation to intracellular calcium influx, leading to pulmonary damage. Mitochondrial function is another important event involved in both vascular diseases and in NP-induced toxicity. Mitochondria are known to provide energy required by cells (ATP) to control apoptosis, to participate in calcium regulation, and in ROS production [59]. Moreover, mitochondrial alterations have also been evidenced in pulmonary arteries in PH [60]. Metal exposure, such as cadmium exposure, induces a decrease in ATP production and an increase in ROS production, resulting in epigenetic modifications and leading to mitochondrial dysfunction associated with the development or aggravation of PH [61]. Mitochondrial O_2_^−^ production might induce a mitochondrial membrane lipoperoxidation, disrupt the ΨMP and activate apoptosis [62]. Previous studies also reported that mitochondrial impairment may contribute to endothelial dysfunction and cardiovascular diseases [63,64]. Finally, we previously demonstrated that NiONPs can activate the apoptosis pathway. Consequently, in the present study, we evaluated the NiONPs effect on mitochondrial function, i.e., ΨMP and mitochondrial mass in both conditions. Our results showed that without NiONPs, mitochondria had more impaired function under pathological conditions with an excessive loss of ΨMP and a decreased in mitochondrial mass, which could also exacerbate PH. In cells under pathological conditions, insignificant amplification of the alteration in both ΨMP and mitochondrial mass after NiONPs exposure was observed as compared to cells under physiological conditions. These results do not seem surprising, since without NiONPs, in cells under pathological conditions mitochondria were already greatly altered in this model mimicking PH. Abnormal mitochondrial dynamics could be an early marker of PH and monitor disease progression [65]. Furthermore, the change in mitochondrial mass would play an important role in controlling the apoptosis pathway via caspase activation and could thus generate cancer cells [66].

In recent years, particular attention has been paid to co-morbidities in order to investigate the factors contributing to pulmonary and cardiovascular disease exacerbations, such as chronic obstructive pulmonary disease and PH [67]. The present study provides evidence that NiONPs-induced toxic effects are strengthened under pathological conditions that mimic the endothelial dysfunction observed in PH. These data could suggest that workers and other populations inhaling NiONPs and with pre-existing cardiovascular pathology are more susceptible to aggravating their disease.

## 5. Conclusions

The present study shows that, under pathological conditions, NiONPs induce oxidative stress, inflammation, calcium alterations, and mitochondrial dysfunction, as well as significant nitrite production. All these effects are enhanced under pathological conditions as compared to physiological ones. Therefore, it can be assumed that workers and the populations exposed to NiONPs are at risk of exacerbating pre-existing vascular disease.

## Data Availability

Data is contained within the article.
